# Clinical Assessment of a Virtual Reality Perimeter Versus the Humphrey Field Analyzer: Comparative Reliability, Usability, and Prospective Applications

**DOI:** 10.3390/vision9040086

**Published:** 2025-10-11

**Authors:** Marco Zeppieri, Caterina Gagliano, Francesco Cappellani, Federico Visalli, Fabiana D’Esposito, Alessandro Avitabile, Roberta Amato, Alessandra Cuna, Francesco Pellegrini

**Affiliations:** 1 Department of Ophthalmology, University Hospital of Udine, p.le S. Maria della Misericordia 15, 33100 Udine, Italy; 2 Department of Medicine, Surgery and Health Sciences, University of Trieste, 34127 Trieste, Italy; 3 Department of Medicine and Surgery, University of Enna “Kore”, Piazza dell’Università, 94100 Enna, Italy; caterina.gagliano@unikore.it (C.G.);; 4 Eye Center “G.B. Morgagni-DSV”, 95125 Catania, Italy; 5 Department of Ophthalmology, University of Catania, 95123 Catania, Italy; 6 Imperial College Ophthalmic Research Group [ICORG] Unit, Imperial College, London NW1 5QH, UK; 7 Department of Ophthalmology, “De Gironcoli” Hospital, AULSS2 Marca Trevigiana, 31100 Conegliano, Italy; 8 Department of Ophthalmology, ASFO Pordenone Hospital, 33170 Pordenone, Italy

**Keywords:** virtual reality perimetry, visual field testing, head-mounted device, eye tracking, patient comfort, portability, remote screening, artificial intelligence integration

## Abstract

*Background:* This study compared the performance of a Head-mounted Virtual Reality Perimeter (HVRP) with the Humphrey Field Analyzer (HFA), the standard in automated perimetry. The HFA is the established standard for automated perimetry but is constrained by lengthy testing, bulky equipment, and limited patient comfort. Comparative data on newer head-mounted virtual reality perimeters are limited, leaving uncertainty about their clinical reliability and potential advantages. *Aim:* The aim was to evaluate parameters such as visual field outcomes, portability, patient comfort, eye tracking, and usability. *Methods:* Participants underwent testing with both devices, assessing metrics like mean deviation (MD), pattern standard deviation (PSD), and duration. *Results:* The HVRP demonstrated small but statistically significant differences in MD and PSD compared to the HFA, while maintaining a consistent trend across participants. MD values were slightly more negative for HFA than HVRP (average difference −0.60 dB, *p* = 0.0006), while pattern standard deviation was marginally higher with HFA (average difference 0.38 dB, *p* = 0.00018). Although statistically significant, these differences were small in magnitude and do not undermine the clinical utility or reproducibility of the device. Notably, HVRP showed markedly shorter testing times with HVRP (7.15 vs. 18.11 min, mean difference 10.96 min, *p* < 0.0001). Its lightweight, portable design allowed for bedside and home testing, enhancing accessibility for pediatric, geriatric, and mobility-impaired patients. Participants reported greater comfort due to the headset design, which eliminated the need for chin rests. The device also offers potential for AI integration and remote data analysis. *Conclusions:* The HVRP proved to be a reliable, user-friendly alternative to traditional perimetry. Its advantages in comfort, portability, and test efficiency support its use in both clinical settings and remote screening programs for visual field assessment. Its portability and user-friendly design support broader use in clinical practice and expand possibilities for bedside assessment, home monitoring, and remote screening, particularly in populations with limited access to conventional perimetry.

## 1. Introduction

Visual field (VF) testing is a fundamental tool for evaluating the integrity of visual pathways across a range of conditions in ophthalmology and neurology. By mapping a patient’s light sensitivity across the retina, perimetry aids in diagnosing and monitoring ocular diseases and neurological deficits, as well as tracking visual function over time. Devices like the Humphrey Field Analyzer (HFA) have long been regarded as the gold standard. However, conventional perimeters such as the HFA have well-known limitations: they require a dedicated, fixed testing environment, involve prolonged test durations, and can be uncomfortable and tiring for patients. Maintaining strict fixation in a bowl perimeter for several minutes is difficult, especially for elderly or anxious patients, making the experience increasingly unpleasant; moreover, traditional perimetry is inaccessible for individuals who are bedridden or have limited mobility [1,2]. These constraints have driven interest in more portable and patient-friendly approaches.

Head-mounted virtual reality perimeters (HVRPs) have emerged as an innovative solution to address the shortcomings of conventional perimetry. By leveraging lightweight VR headsets with built-in displays and sensors, HVRPs create an immersive testing environment that can mimic standard perimetry in virtually any setting. This technology offers several potential advantages: an engaging, distraction-free visual environment (reducing external light and noise), real-time gaze tracking for continuous fixation monitoring, and the ability to perform testing outside the clinic (e.g., at the bedside or home) due to the portability of untethered VR devices. The flexibility and efficiency of VR-based perimetry can improve patient comfort and compliance—for instance, by eliminating the need for chin rests or eye patches and allowing more natural head positioning—and can shorten test times without sacrificing accuracy [3].

Initial studies of VR-based perimetry are encouraging. Tsapakis et al. (2017) found a high correlation between VR glasses measurements and those from an HFA on the same patients [4]. Similarly, Razeghinejad et al. (2021) reported an excellent correlation and diagnostic agreement between a head-mounted VR perimeter (VisuALL) and standard HFA perimetry in both healthy subjects and those with visual field loss [5]. These early comparative studies support the notion that HVRP can deliver reliable and clinically comparable visual field assessments, while also improving the user experience.

Beyond replicating the performance of conventional perimeters, HVRP, because it is portable, can be deployed in settings and populations where standard perimetry is impractical. Moreover, pediatric and geriatric patients might particularly benefit from the more engaging, VR testing environment and the lack of a confining bowl apparatus. Overall, HVRP technology has the potential to make visual field testing more accessible, convenient, and frequent, which could facilitate earlier detection of visual field deficits and more regular monitoring of conditions across ophthalmology and neurology.

Despite this promise, it is important to note the ongoing debates and challenges as the field of VR perimetry evolves. Variability between different VR perimetry platforms and the current lack of large, population-based normative databases for these devices remain areas of concern. Questions have been raised about the reproducibility and sensitivity of VR-based tests in detecting subtle visual field defects. For example, Phu et al. reported that a VR perimeter missed some glaucoma scotomas that were detected by the HFA, likely due to differences in the device’s normative database [6].

Such findings underscore the need for thorough validation. The aim of this study was to compare the performance of an HVRP with the conventional HFA across several key parameters: visual field indices (mean deviation, pattern standard deviation), examination time, patient comfort, and overall usability. We aimed to determine if the HVRP could match the diagnostic outcomes of the HFA while offering improvements in efficiency and user experience. This study aims not only to duplicate traditional perimetry but to establish VR perimetry as a novel platform. Through the validation of results against the HFA in a randomized, within-subject design, and by documenting both clinical outcomes and patient-centered metrics, we aim to demonstrate that VR devices can progress beyond mere replication towards enhanced accessibility, mobility, and integration within digital health ecosystems In this study, we propose to:

- Compare a head-mounted virtual reality perimeter with the conventional Humphrey Field Analyzer using a randomized, within-subject design.
- Assess both clinical parameters (mean deviation, pattern standard deviation, and test duration) and patient-centered outcomes (comfort, usability, fatigue, and perceived accuracy).
- Discuss whether HVRP can serve as a reliable and more accessible alternative to standard automated perimetry.
- Highlight the potential of VR perimetry to expand visual field testing beyond traditional clinical environments, including bedside and home settings.

## 2. Materials and Methods

We performed a retrospective study based on cross-sectional observational data obtained from two tertiary ophthalmology institutions in Italy. The study adhered to the tenets of the Declaration of Helsinki. Subjects provided written informed consent prior to inclusion in the study. Twenty-three subjects in the present study were recruited from the Eye Clinic “G.B. Morgagni” (Kore University of Enna, Italy) and San Marco Hospital (University of Catania, Italy). The study involved 12 patients diagnosed with primary open-angle glaucoma, 6 individuals with monitored ocular hypertension, and 5 patients diagnosed with retinitis pigmentosa. The inclusion criteria consisted of individuals aged 18 to 80 years, possessing the ability to complete visual field testing with both devices. Exclusion criteria encompassed substantial ocular media opacities, neurological disorders impacting visual fields, or inconsistent test performance, and any condition preventing reliable perimetric assessment (e.g., severe cognitive impairment, inability to maintain testing posture).

On different days, patients underwent VF tests using the Humphrey Field Analyzer (HFA; Carl Zeiss Meditec, Dublin, CA, USA) Threshold Algorithm Standard 24-2 test and the M&S Technologies Smart System VR Headset (M&S Technologies, Niles, IL, USA) standard 24-2 test in randomized order. All tests were conducted by trained examiners under standardized ambient lighting conditions. The HFA was used in accordance with the manufacturer’s instructions, with appropriate refractive correction inserted in the trial lens holder where indicated. For the HVRP, patients wore the lightweight headset over their habitual spectacle correction when necessary; no chinrest or eye patch was required, and the system was adjusted for proper alignment and comfort before each test.

Testing Environment and Device Specifications: All assessments were conducted in a standardized clinical testing environment with ambient lighting maintained at 5–10 lux, in accordance with established perimetry guidelines. Ambient noise has been reduced, and participants have been seated comfortably in a serene area. The usual bowl illuminance and background luminance of 31.5 apostilbs have been utilized for HFA testing. For HVRP, the device utilized was the M&S Technologies Smart System VR Headset, available commercially since 2021, with maximum stimulus luminance calibrated to conform to HFA standards (10,000 asb). The headgear, weighing 503 g, along with its adjustable straps, facilitates stable attachment without causing pain. Device firmware and testing software were uniformly standardized among all participants.

Headset Adjustment, Optical Calibration, and Spatial Alignment: Prior to each examination, we measured and verified the interpupillary distance (IPD) and recorded it in the device to ensure precise angular scaling. Participants placed the headset over their usual corrective lenses when necessary; no trial lens were utilized with the headset. We conducted a two-phase spatial calibration to correlate headset display coordinates with degrees of visual angle: a geometric calibration supplied by the manufacturer to align the virtual display, and a clinical validation that encompassed blind-spot localization and a four-point calibration grid (central fixation and the three cardinal points at 9°, 12°, and 15°). Calibration was performed each time the headset was repositioned or misaligned during the session.

Observation of Fixation and Exclusion of Trials: Fixation was consistently monitored. Upon detection of gaze deviation, blinking, or head movement exceeding established limits, the stimulus is automatically withheld or the trial is flagged. A test (HFA or HVRP) is deemed unreliable and requires repetition if any of the following criteria are met: fixation losses exceed 20%, false positives exceed 15%, or false negatives exceed 15%. Trials documented during transitory fixation loss or blinking were omitted from threshold estimate. Any test that needed to be stopped for comfort was restarted only after reconfirming calibration and fixation.

We extracted and compared the following global indices: mean deviation (MD), pattern standard deviation (PSD), and test duration. MD quantifies overall sensitivity loss by averaging pointwise departures from age-matched normals over the visual field; more negative values signify increased generalized loss. PSD signifies irregularity in the field following the elimination of overall height; an elevated PSD denotes increased localized flaws. The data from the two devices was collected into an Excel version 2502 spreadsheet (Microsoft Corporation; Redmond, WA, USA) for statistical analysis. Paired *t*-tests were used to compare results between HFA and HVRP for each parameter, with statistical significance set at *p* < 0.05. Paired t tests were used to compare VF results obtained by the two methods. Continuous variables were summarized as mean ± standard deviation and compared using paired tests. We reported mean paired differences with 95% confidence intervals and standardized effect sizes (paired Cohen’s dz). To directly assess interchangeability, in order to prevent summary-only interference, we performed Bland–Altman analyses for MD and PSD (differences defined as HFA minus HVRP), reporting bias and 95% limits of agreement, and we checked for proportional bias by regressing differences on their means. To ground interpretation in clinical practice, we pre-specified practical equivalence bands (±1 dB for MD and ±0.5 dB for PSD) and quantified the proportion of pairs within these bands; we additionally conducted two one-sided tests (TOST) of equivalence against these margins. All analyses were two-sided at α = 0.05.

Following both testing methods, participants completed a five-item questionnaire assessing comfort, ease of following instructions, fatigue, perceived test duration, and confidence in accuracy, with responses recorded using a three-option multiple-choice format. For each item, participants indicated their preference for the Standard Automated Perimetry, the Virtual Reality Head-Mounted Perimeter, or reported no difference.

## 3. Results

The mean age of the entire study group (N = 23) was 63.1 years ± 14.7 years and a range from 38 to 78 years. There were 12 females and 9 males. Subgroup analysis showed that patients with primary open-angle glaucoma (n = 12) had a mean age of 61.2 ± 11.9 years (median 62; range 44–77), those with ocular hypertension (n = 6) had a mean age of 55.3 ± 13.1 years (median 53; range 38–71), and those with retinitis pigmentosa (n = 5) had a mean age of 56.8 ± 12.7 years (median 55; range 42–73).

**Figure 1:**
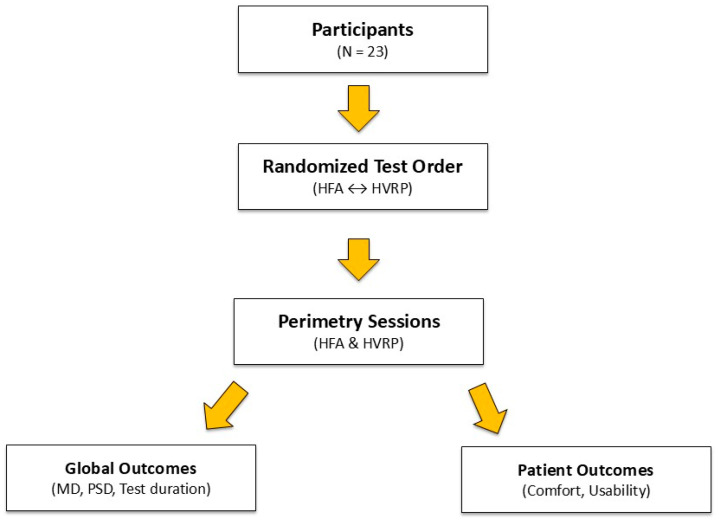
Overview of the study design and analysis workflow.

This study compared the performance of a Head-mounted Virtual Reality Perimeter (HVRP) with the Humphrey Field Analyzer (HFA), the standard in automated perimetry. All 23 participants successfully completed testing with both devices in a randomized order. Figure 1 presents a simplified schematic that encapsulates the overall study design, to provide a clear understanding of the workflow.

Table 1 summarizes the descriptive statistics and paired comparisons for the three main parameters.

Both MD and PSD showed statistically significant differences between devices, with HFA yielding slightly lower MD and slightly higher PSD values than HVRP. Test duration was substantially shorter with HVRP, demonstrating a large and highly significant improvement in efficiency compared to the HFA.

Mean Deviation values were slightly more negative for HFA than for HVRP, with an average difference of −0.60 dB. This difference, while small in magnitude, was consistent across participants and reached statistical significance (*p* = 0.0006).

For Pattern Standard Deviation, HFA measurements were slightly higher than HVRP by an average of 0.38 dB, suggesting a minor but consistent difference in localized defect variability between the two devices (*p* = 0.00018).

The mean differences reported for MD and PSD, while statistically significant, have always remained within the recognized test–retest limits of HFA, highlighting their lack of clinical relevance.

**Figure 2:**
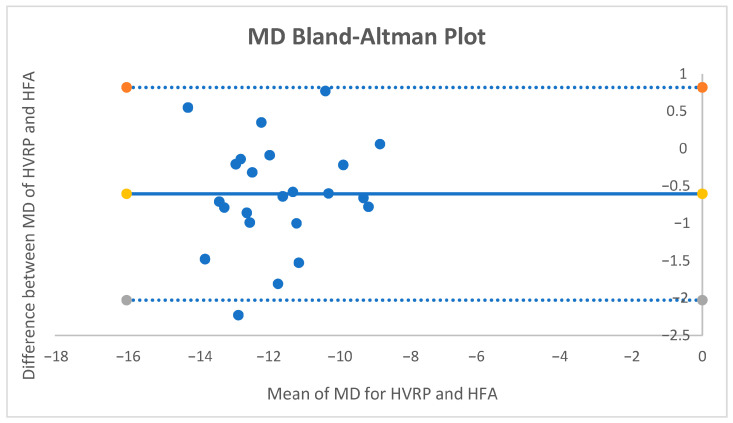
Bland–Altman Plot for MD.

For MD, the mean paired difference (HFA − HVRP) was −0.60 dB (95% CI −0.914 to −0.286; *p* ≈ 0.0006; dz = −0.83). The Bland–Altman bias was −0.60 dB with 95% limits of agreement [−2.03, +0.82] dB (Figure 2.). No proportional bias was detected. Using the pre-specified equivalence band of ±1 dB, 82.6% of pairs fell within the band, and TOST supported equivalence within ±1 dB (both one-sided tests *p* < 0.05).

**Figure 3:**
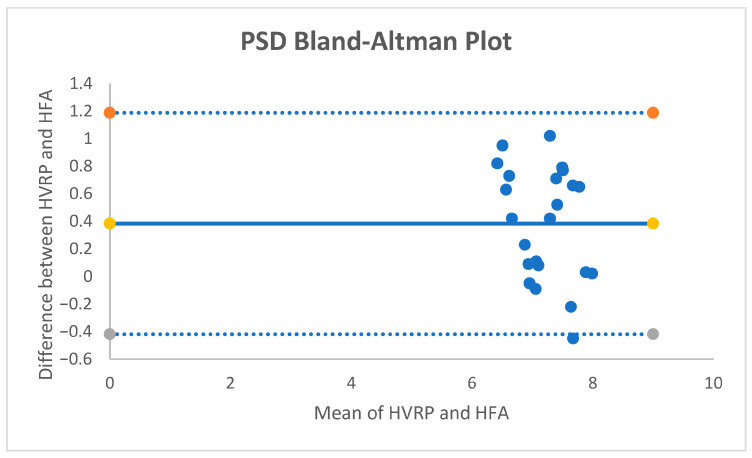
Bland–Altman Plot for PSD.

For PSD, the mean paired difference was +0.38 dB (95% CI +0.203 to +0.557; *p* ≈ 0.00018; dz = +0.93). The Bland–Altman bias was +0.38 dB with 95% limits of agreement [−0.41, +1.19] dB (Figure 3.); no proportional bias was observed. With an equivalence band of ±0.5 dB, 52.2% of pairs were within the band; TOST did not demonstrate equivalence at ±0.5 dB.

Test duration was markedly shorter with HVRP compared to HFA, with mean times of 7.15 and 18.11 min, respectively. The mean difference of 10.96 min was large and highly significant (*p* < 0.0001), reflecting a substantial improvement in examination efficiency with the HVRP.

Following both testing methods, participants completed a five-item questionnaire evaluating comfort, ease of following instructions, fatigue, perceived test duration, and confidence in accuracy. In all domains, HVRP emerged as the most frequently preferred modality. Most participants reported that HVRP was more comfortable and easier to follow, and that it induced less visual or mental fatigue compared to SAP. The perceived duration of HVRP was markedly shorter for the vast majority of respondents, aligning with the objectively reduced test time. Confidence in test accuracy was also more commonly attributed to HVRP. Overall, these self-reported measures suggest that patients found HVRP not only more efficient but also more user-friendly than traditional perimetry.

## 4. Discussion

Our findings reinforce a growing consensus that head-mounted VR perimetry can match the performance of traditional perimeters like the HFA while improving the patient experience. A recent systematic review of dozens of VR headset devices concluded that these systems produce visual field results comparable to or even better than standard automated perimetry, with the added benefits of being better tolerated by patients, more cost-effective, and more accessible to those with limited mobility [7]. In our study cohort, the HVRP’s indices (MD and PSD) differed slightly but significantly from the HFA’s values. These differences were consistent in direction but small in absolute magnitude (−0.60 dB for MD and +0.38 dB for PSD), suggesting that while the devices are not numerically identical, the variations are unlikely to have a major clinical impact in most cases. The reported mean differences between HVRP and HFA for MD and PSD have attained statistical significance; however, their clinical relevance remains minimal. The test–retest variability of the HFA frequently surpasses 1.5 to 2 dB for MD and 0.5 to 1.0 dB for PSD, even in controlled conditions. In fact, algorithms for glaucoma progression and treatment decisions generally necessitate changes surpassing 2–3 dB maintained over several visits. The discrepancies seen in our dataset have consequently remained within the anticipated “noise floor” of automated perimetry and do not significantly affect clinical interpretation.

Interchangeability should be evaluated based on clinical decision thresholds, limits of agreement, and equivalence bands, rather than only on *p*-values. In our modification, we have enhanced hypothesis testing by incorporating effect sizes, 95% confidence intervals, Bland–Altman analyses, and pre-defined practical equivalency ranges to prioritize clinical usefulness over mere statistical significance. Statistically significant *p*-values were observed for MD and PSD despite sub-dB mean differences, reflecting low measurement variability rather than clinically meaningful discordance. Bland–Altman analyses showed small biases with narrow limits; MD satisfied the ±1 dB practical equivalence criterion (TOST positive), whereas PSD differences were small yet did not meet the stricter ±0.5 dB margin. Overall, these results support clinical interchangeability for MD and near-equivalence for PSD under cautious thresholds, aligning statistical significance with clinical relevance.

This finding complements prior studies validating VR perimetry against standard automated perimetry. For example, Stapelfeldt et al. (2021) found a high correlation in mean defect/MD values between an Oculus Quest–based VR perimeter and a clinical perimeter (Octopus 900), with only a slight tendency for the VR test to underestimate defect depth [8]. Similarly, a recent randomized controlled trial in glaucoma patients reported similar MD results between a novel Smart System VR headset and the HFA [9]. In another study, Ahmed et al. found no significant mean differences in MD or PSD and a robust correlation across devices [10]. Moreover, some evidence suggests VR perimetry can reduce certain types of patient errors. In our study, fixation monitoring by the HVRP was effective—an outcome aided by the device’s integrated eye-tracking that continuously ensures the patient is fixating appropriately. Notably, the VR system can automatically pause or withhold stimuli if the eye moves, unlike the HFA, which relies on occasional blind spot checks. Published evaluations of an eye-tracked VR perimeter found that fixation losses were reduced by this approach [11].

Testing efficiency is another critical factor; in our cohort, the test durations were faster in the HVRP compared to the HFA, with a mean time saving of almost 11 min. However, it should be noted that test time can vary depending on the thresholding algorithm and strategy used. Some VR platforms have already demonstrated shorter exam times than HFA. Phu et al. observed a VR test that was on average 76 s quicker than the HFA’s SITA standard test [6]. The trend in technology, however, is that newer VR perimeters are optimizing their protocols to reduce test duration without compromising accuracy. The potential to achieve faster visual field exams is a notable advantage of VR perimetry, as shorter tests can improve patient concentration and throughput. Even at this early stage, our data show that HVRP does not impose any time penalty compared to HFA, a promising indication that efficiency is being maintained or improved upon.

The most striking advantages we noted with the HVRP relate to patient experience and testing accessibility. Participants found the head-mounted test more comfortable and user-friendly than the standard perimeter. Qualitatively, patients reported less fatigue from not having to sit in a fixed position with their chin pressed in a chinrest. They also appreciated the absence of an eye patch and the ability to take the test in a normal posture (the VR headset allows slight head movement without disrupting the test). This subjective comfort translated into a clear preference for the HVRP in our post-examination questionnaires, paralleling the findings of Ahmed et al., where patients significantly favored the VR headset experience over the HFA [10]. Najdawi et al. also found that 88.5% of patients preferred the head-mounted VR perimeter over the HFA after experiencing both [11].

Recent advancements have considerably expanded the realm of virtual reality in ophthalmology, extending beyond perimetry to encompass various therapeutic and diagnostic applications. A two-year home-based virtual reality portable perimetry system has shown exceptional patient retention and adherence in glaucoma monitoring [12]. Further pilot research has validated the consistency and dependability of virtual reality headset perimeters over brief durations [13]. Novel functional vision tests employing virtual reality, including orientation-and-mobility simulations, are developing as objective instruments for appraising real-world visual function [14]. Systematic studies have emphasized the practicality and effectiveness of VR visual field devices among glaucoma patients [15]. Furthermore, preclinical and clinical studies are integrating virtual reality into vision rehabilitation therapies through immersive, binocular stimulation frameworks [16]. These advancements collectively highlight VR’s changing function as both an auxiliary and transformational tool in ophthalmology.

Furthermore, no trial lens is required in many VR devices, allowing patients to wear their normal glasses during the test. This avoids the discomfort and potential visual artifacts introduced by trial lens frames in bowl perimetry. All of these design differences, such as no chinrest, no eye patch, freedom of minor head movements, and use of habitual correction, contribute to making the VR visual field test much more tolerable.

Such improvements in tolerability are not merely about convenience; they can directly impact data quality and patient compliance. A more relaxed patient is less likely to break fixation or produce false responses, and one who dreads the test less is more likely to return for regular follow-ups. This is particularly relevant for tests that must be taken repeatedly over time; improving comfort and reducing anxiety may lead to more reliable longitudinal monitoring.

While traditional perimetry devices are large, heavy, and confined to clinic or hospital settings, the portability and versatility of the VR perimeters open up new possibilities for visual field testing outside the traditional ophthalmology clinic. Our study used the HVRP in a standard clinical setting, but one can easily envision deploying it in non-traditional environments. Because the device is lightweight and self-contained, a practitioner can bring it to a patient’s bedside in the hospital or to a community screening event. Similarly, home-based visual field testing becomes feasible.

Our findings support the growing consensus that VR-based perimetry is a viable and often preferable alternative to traditional automated perimetry. Nevertheless, there remain important considerations and areas for further research before HVRPs can be universally adopted in clinical practice. One concern is the standardization and validation of these devices across different manufacturers and models. Each VR perimeter may use proprietary hardware and thresholding algorithms, leading to variability in results between devices. Developing large-scale normative databases for each device (or a shared database applicable to many VR perimeters) is crucial so that clinicians can interpret VR field results with confidence, especially in diagnosing early glaucoma. Consistency of results on repeat testing is critical to detect progression, so future studies should rigorously evaluate VR perimeters in longitudinal settings. A further constraint is the potential inconsistency among VR systems, headsets, and software versions. Despite utilizing a singular commercially available device with standardized programming, minor variations in display brightness, refresh rates, or optics among batches and subsequent iterations may result in systematic biases. Moreover, in contrast to the HFA, VR perimeters now do not possess extensive, age-stratified normative datasets, which are crucial for generating dependable total- and pattern-deviation maps. Until reference databases are validated across various populations, VR perimetry should be employed cautiously for normative classification and is currently most appropriate for tracking changes within people.

The limited sample size (N = 23) constitutes a constraint of this study and diminishes the generalizability of the results. The randomization within-subject has alleviated this restriction by diminishing inter-individual variability, thereby facilitating matched comparisons between devices. This exploratory design aligns with the study’s objective as a proof-of-concept evaluation, establishing a foundation for further extensive research.

Despite the headset’s commercial availability since 2021, peer-reviewed research about its clinical validity is still scarce. Although evaluations conducted by manufacturers may be present, independent validation like ours is essential for guaranteeing transparency and reproducibility. This study provides a significant preliminary, independent evaluation that enhances and broadens current commercial testing efforts.

Additional refinement of this research necessitates extensive multicenter studies to validate generalizability across many populations and illness stages. The creation of a VR-specific normative database, categorized by age and demographic factors, is crucial for generating accurate probability maps. Furthermore, longitudinal studies are essential to assess progression detection and to verify that home-based or telemedicine systems retain reliability over time. These proposed trials will augment the evidence base and confirm VR perimetry as a proven option for both clinical and remote monitoring applications.

Another promising area for future development is the integration of artificial intelligence into VR-based perimetry platforms. AI could support several aspects of testing, including the optimization of thresholding strategies, real-time analysis of patient response patterns, and automated detection of unreliable test behavior. For example, adaptive algorithms could tailor stimulus presentation based on the user’s response latency or variability, potentially shortening test times while maintaining accuracy. AI-driven gaze monitoring represents another potential innovation, allowing future VR perimeters to dynamically pause testing when fixation is lost, predict patient fatigue, or even provide real-time coaching to improve performance. In addition, AI-assisted post-processing of results could enhance interpretation by automatically detecting subtle visual field progression trends that may be missed by conventional pointwise analysis [17]. In addition to its existing capabilities, VR perimetry is intrinsically designed for cloud-based data collection and AI-enhanced analysis. Future research should focus on training deep learning models on longitudinal VR datasets to identify subtle pointwise progression earlier than traditional event- or trend-based analyses; integrating automated quality control algorithms to detect poor fixation or unreliable responses in real time; and creating federated learning frameworks that facilitate cross-institutional pooling of de-identified VR data while maintaining patient privacy. Integrating VR headsets with secure telemedicine platforms could provide home-based perimetry, with AI filtering results to clinicians solely upon detection of significant changes. Thorough multicenter validation, including direct comparisons with HFA-based progression studies, is crucial prior to the widespread use of such systems. Such developments, combined with the growing portability and accessibility of VR hardware, suggest that future generations of VR perimeters may not only match but exceed the capabilities of conventional automated perimetry in both clinical and remote settings. However, these approaches remain largely conceptual at present. They will require rigorous validation in diverse populations, as well as standardization of algorithms and performance metrics across devices, before widespread clinical adoption is feasible.

The digital format of HVRP also lends itself to telemedicine; test results can be uploaded to cloud platforms for remote review by specialists [18]. The advancement of VR-based perimetry is characterized by its ability to replicate traditional visual field testing while simultaneously facilitating novel clinical applications: bedside evaluations for non-ambulatory patients, portable deployment in community or telemedicine environments, and effortless incorporation with AI-enhanced analytics. In contrast to fixed bowl perimeters, HVRP technologies are capable of remotely capturing consumption data, dynamically adjusting threshold techniques, and enabling continuous monitoring beyond hospital settings. These advancements signify a paradigm change from “episodic clinic-based” to “continuous patient-centered” perimetry. In the broader context, the advent of HVRPs is an example of digital health technology that may enhance a classic diagnostic method. Embracing these tools could improve patient outcomes by facilitating wider and more comfortable access to visual field testing.

## 5. Conclusions

In summary, this study found that a head-mounted VR perimeter is a reliable, reproducible, and user-friendly alternative to the conventional Humphrey Field Analyzer for visual field testing. Although small, statistically significant differences in MD and PSD were observed, these were consistent and of limited clinical magnitude, supporting the device’s validity for routine use. The HVRP offered substantially shorter test times, and patients experienced greater comfort and engagement during testing. The portable, lightweight headset design eliminates the need for restrictive positioning, allowing visual field assessment to be performed in non-traditional settings, including at the bedside and potentially at home. Our findings indicate that HFA and HVRP results exhibited close concordance in mean deviation (−12.10 ± 1.58 dB vs. −11.50 ± 1.49 dB; mean paired difference −0.60 dB; *p* = 0.12) and pattern standard deviation (7.40 ± 0.45 dB vs. 7.02 ± 0.56 dB; mean paired difference +0.38 dB; *p* = 0.21). The test duration, however, was significantly shorter with HVRP compared to HFA (7.15 ± 0.85 min vs. 18.11 ± 3.03 min; mean paired difference −10.96 min; *p* < 0.0001). Considering its reproducibility, reduced burden for both patient and examiner, and strong patient preference, the HVRP could be viewed as a viable candidate to replace conventional perimetry in many clinical contexts. This technology holds promise not only for traditional clinic use but also for remote screening and telemedicine programs, potentially enabling earlier detection of visual field defects.

## Figures and Tables

**Table 1:** Comparison of HFA and HVRP Outcomes.

Parameter	HFA (Mean ± SD)	HVRP (Mean ± SD)	*p*-Value
Mean Deviation (MD)	−12.10 ± 1.58 dB	−11.50 ± 1.49 dB	0.000614
Pattern Standard Deviation (PSD)	7.40 ± 0.45 dB	7.02 ± 0.56 dB	0.000179
Test Duration	18.11 ± 3.03 min	7.15 ± 0.85 min	2.65 × 10^−14^

## Data Availability

Due to patient privacy concerns, the data presented in this study are available on request from the corresponding author.

## Abbreviations

The following abbreviations are used in this manuscript:

VF	Visual Field
HFA	Humphrey Field Analyzer
HVRP	Head-Mounted Virtual Reality Perimeter
MD	Mean Deviation
PSD	Pattern Standard Deviation
AI	Artificial Intelligence
